# 17β-Estradiol Protects Human Eyelid-Derived Adipose Stem Cells against Cytotoxicity and Increases Transplanted Cell Survival in Spinal Cord injury

**DOI:** 10.1111/jcmm.12191

**Published:** 2013-12-22

**Authors:** Jing Zhou, Ping Lu, Hao Ren, Zefeng Zheng, Junfeng Ji, Hua Liu, Fangzhen Jiang, Shucai Ling, Boon Chin Heng, Xueqing Hu, HongWei Ouyang

**Affiliations:** a Center for Stem Cell and Tissue Engineering, School of Medicine, Zhejiang UniversityHangzhou, China; b Zhejiang Provincial Key Laboratory of Tissue Engineering and Regenerative MedicineHangzhou, China; c Institute of Anatomy and Cell Biology, Medical College, Zhejiang UniversityHangzhou, China; d Department of Plastic Surgery of 2nd Affliated Hospital, School of Medicine, Zhejiang UniversityHangzhou, China; e Department of Biosystems Science & Engineering (D-BSSE), ETH-ZurichBasel, Switzerland; f Collaborative Innovation Center for Diagnosis and Treatment of Infectious DiseasesHangzhou, China

**Keywords:** cell transplantation, spinal cord injury, hEASCs, 17-β-estradiol

## Abstract

Stem cell transplantation represents a promising strategy for the repair of spinal cord injury (SCI). However, the low survival rate of the grafted cells is a major obstacle hindering clinical success because of ongoing secondary injury processes, which includes excitotoxicity, inflammation and oxidative stress. Previous studies have shown that 17b-estradiol (E2) protects several cell types against cytotoxicity. Thus, we examined the effects of E2 on the viability of human eyelid adipose-derived stem cells (hEASCs) *in vitro* with hydrogen peroxide (H_2_O_2_)-induced cell model and *in vivo* within a rat SCI model. Our results showed that E2 protected hEASCs against H_2_O_2_-induced cell death *in vitro*, and enhanced the survival of grafted hEASCs *in vivo* by reducing apoptosis. Additionally, E2 also enhanced the secretion of growth factors by hEASCs, thereby making the local microenvironment more conducive for tissue regeneration. Overall, E2 administration enhanced the therapeutic efficacy of hEASCs transplantation and facilitated motor function recovery after SCI. Hence, E2 administration may be an intervention of choice for enhancing survival of transplanted hEASCs after SCI.

## Introduction

Spinal cord injury (SCI) is one of the most devastating forms of traumatic injury, often resulting in permanent disability 1. The repair of SCI is still a major therapeutic challenge at present, mainly because of the limited endogenous repair capacity of the mammalian central nervous system. Therefore, exogenous intervention strategies are necessary to facilitate patient recovery after SCI 2. Although there are currently no universally accepted and effective treatment modality for this traumatic disorder, cytotherapy may provide a promising alternative therapeutic strategy for SCI. Several cell types have been evaluated as potential seed cells for SCI transplantation, such as embryonic stem cells 3, neural precursor cells 4, mesenchymal stem cells (MSCs) 5, olfactory ensheathing cells 6 and Schwann cells 7. The goals of cell transplantation therapy vary widely and include replacing damaged neurons, filling the cystic cavity, creating a regenerative environment and supporting remyelination 7. However, the limited availability of some cell types, low repair efficacy and ethical concerns have necessitated evaluation of other alternative candidate cell types for SCI therapy.

Previous studies have reported that novel stem cells derived from human eyelid adipose tissue possessed neural crest characteristics. These are widely referred to as human eyelid adipose-derived stem cells (hEASCs) 8. Unlike MSCs derived from other adipose tissues, hEASCs originate from the neural crest source, and express markers associated with human multi-potent neural crest cells 8,9. Furthermore, hEASCs are easily accessible and free of ethical complications unlike human embryonic and foetal stem cells. Hence, it is possible that hEASCs may be an excellent candidate therapeutic cell type for SCI therapy.

Nevertheless, a major obstacle hindering successful stem cell therapy of SCI is the poor survivability of transplanted cells because of ongoing secondary injury processes, which includes excitotoxicity, inflammation and oxidative stress 10,11. Indeed, grafted cells are particularly sensitive to oxidative stress as indicated by studies of neuropathy, in which oxidative stress is a critical component. Various strategies to improve the survival of transplanted cells have been proposed, such as the induction of neurotropic factors and activation of anti-apoptotic genes 10,12. In early treatment of SCI, various pharmacologically active agents have been shown to halt the spread of secondary tissue damage, maximizing the extent of undamaged neurological tissue and curbing inflammation 13,14. Amongst the various types of small molecule drugs tested, oestrogen has demonstrated a robust cytoprotective effect against oxidative damage, inflammation and apoptosis^15^–^19^ in diverse cell types including neuronal cell lines, primary neuronal cells, hepatocytes, fibroblasts and oligodendrocytes, both *in vitro and vivo*^20^–^23^. Moreover, recent studies have demonstrated that post-SCI administration of 17-β-estradiol (E2) decreased lesion volume, and reduced secondary damage and attenuated apoptosis following SCI 18,24–26. However, it remains to be determined whether E2 administration can support the survival of cells against oxidative damage *in vitro* and *in vivo*.

In this study, we suggest that E2 has a cytoprotective effect on hEASCs against oxidative stress *in vitro* and that the combination of E2 administration and hEASCs transplantation after SCI in a rat model will increase hEASCs survival *in vivo* and improve the functional recovery of paralyzed animals in a rat SCI model. These findings may have implications that E2 administration may be an intervention of choice for enhancing survival of transplanted hEASCs after SCI.

## Materials and methods

### Human eyelid adipose-derived stem cell isolation and culture

Human eyelid adipose samples were obtained with informed consent from four patients aged between 20 and 30 years undergoing eyelid cosmetic surgery, at the Second Affiliated Hospital of Zhejiang University. All experiments were approved by the Institutional Review Board of Zhejiang University. Adipose tissues were surgically dissected from the subcutaneous zone, cut into 1–2 mm^3^ pieces and washed three times with PBS. The tissue fragments were digested with 0.25% collagenase (Sigma-Aldrich Inc., St. Louis, MO, USA) overnight at 37°C. Following centrifugation at 1250 *g* for 10 min., cell pellets were isolated and washed twice in DMEM-low glucose type (DMEM-LG; Gibco-BRL Inc., Grand Island, NY, USA). Cell suspensions were cultured in DMEM-LG, supplemented with 10% foetal bovine serum (FBS; Gibco) and 1% penicillin–streptomycin (Gibco) at 5% CO_2_ and 37°C. Fresh medium was replaced every 3 days 8. After 2 weeks in culture, adherent cells were obtained and subjected to serial passage. Cells between passages 2 and 14 were utilized for further studies.

### Monoclonal selection and colony forming unit (CFU) assay

The cells were seeded at very low density (three cells/cm^2^) to form monoclonal colonies and cultured in L-DMEM supplemented with 1% penicillin–streptomycin and 20% FBS. After 10–12 days, the colonies were stained with 1% crystal violet (Sigma-Aldrich) in methanol for 10 min. The number of colonies with diameter >2 mm were counted.

### Fluorescence-activated cell sorting (FACS) analysis

After trypsinization, detached hEASCs were resuspended and 1 × 10^6^ cells were incubated with 1 μg of phycoerythrin (PE) or fluorescein isothiocyanate (FITC)-conjugated mouse anti-human monoclonal antibodies for 1 hr at 4°C. Phycoerythrin-or FITC-conjugated isotype-matched IgGs (BD Biosciences Pharmingen Inc, San Diego, CA, USA) were utilized as controls. After washing, the samples were analysed on a Coulter Epics XL flow cytometer (Beckman-Coulter Inc, Brea, CA, USA). All monoclonal antibodies (Table 1) for flow cytometry analysis were purchased from BD Pharmingen.

**Table 1:** List of antigens examined for the immunophenotyping of hEASCs

Antigen	Other names	Origin of antibody	Function	Catalogue number	Supplier
For FACS analyses					
CD18	Intergrinβ2chain, CR3/CR4	Mouse	Bone marrow stromal cell marker	555923	BD pharmingen
CD34	gp105-120	Mouse	Haematopoietic stem cell marker	555821	BD pharmingen
CD44	Pgp-1,H-CAM, Ly24	Mouse	Mesenchymal stem cell marker	550989	BD pharmingen
CD29	Intergrinβ1chain	Mouse	Mesenchymal stem cell marker	556048	BD pharmingen
CD105	Endoglin	Mouse	Mesenchymal stem cell marker	555690	BD pharmingen
CD166	–	Mouse	Cell adhesion molecule	559260	BD pharmingen
For immunocytochemistry analyses					
GFAP	Glial fibrillary acidic protein	Rabbit	Astrocyte marker	AB5804	Chemicon
NESTIN	–	Purified immunoglobulin	Neural stem cell marker	MAB5326	Chemicon
GAP43	Growth-associated protein 43	Rabbit	Neuron marker	AB5312	Chemicon
GALAC	GALACTOCERE-BROSIDE	Rabbit	Oligodendrocyte marker	AB142	Chemicon
APC	APC(C-TERMINAL)	Purified immunoglobulin	Oligodendrocyte marker	MAB3786	Chemicon
NeuN	NeuN clone 60	Mouse	Neuron marker	MAB377	Chemicon
MAP2	Microtubule associated protein 2	Rabbit	Neuron marker	AB5622	Chemicon
Human Nuclear	–	Mouse	Human Nuclear marker	MAB1281	Chemicon
NF-200	Human Neurofilament 200	Mouse	Neuron marker	MAB353	Chemicon
DOPA	DOPA Decarboxylase	Rabbit	Neuron marker	AB1569	Chemicon
Vimentin	–	Mouse	Mesenchymal stem cell marker	103465-002	Dakocytomation

### Cell proliferation assay

The viability and proliferation of hEASCs were assayed with Cell Counting KIT-8 (CCK-8; Dojindo Laboratories Inc., Kumamoto, Japan). The hEASCs were incubated in CCK-8 solution in a 5% CO_2_ incubator at 37°C for 3 hrs at various time-points (1, 3, 5 and 7 days). The absorbance was measured at 450 nm with a reference wavelength of 650 nm. Cell numbers were correlated with optical density (OD).

### Assessment of multi-potential differentiation

Human eyelid adipose-derived stem cells at passage 4 were utilized to evaluate the multi-potential differentiation of these cells *in vitro* as described previously^27^–^29^. Oil red O staining, alkaline phosphatase (ALP) activity and safranin O staining were employed to assess the adipogenic, osteogenic and chondrogenic differentiation potential of these cells respectively. All data represent mean ± SD of four independent experiments.

### Gene expression profile of hEASCs

Total cellular RNA was isolated from cultured hEASCs using Trizol reagent (Invitrogen, Carlsbad, CA, USA). Reverse transcription (RT) of mRNAs was performed using MMLV Reverse Transcriptase (Ambion Inc, Austin, TX, USA) with poly-dT as primer and with a Mastercycler thermal cycler (Eppendorf Inc, Hamburg, Germany). The pan-neural gene expression profile was analysed (Table 2) using RT-PCR. Human embryonic stem cells (an undifferentiated NIH-registered human ESC H9 WiCell) and human neuroblastoma cells (SH-SY5Y; kindly donated by Prof. Zhengping Xu, Zhejiang University, China) were utilized as controls.

**Table 2:** List of gene primers examined of hEASCs

Gene	Sequence	Size(bp)	Temp (°C)	Number	Species
nestin	AGCTGGCGCACCTCAAGAT	183	60	NM_006617.1	Human
	GCAAAGATCCAAGACGCCG				
mbp	ACCGGCTCATTCACTTCC	226	60	NM_001025101.1	Human
	CAATCACAGGTGCGCTAA				
scf	GTATCAACACTGTTACTTTCG	266	60	NM_000899.4	Human
	TAAATGAGACCCAAGTCCCG				
gap43	AGGCAAGGGACGAGACAACC	151	60	NM_002045.3	Human
	CCACGGAAGCTAGCCTGAAT				
cnpas	GGAGCTGCGACAATTCGTCC	280	60	NM_033133.4	Human
	CACATCACTCGGCCACAACT				
tubb3	TACGTGCCTCGAGCCATTC	153	60	NM_006086.2	Human
	CCCCTCCGTGTAGTGACC				
s100b	CCAATATTCTGGAAGGGAGG	213	60	NM_006272.2	Human
	TCGTGGCAGGCAGTAGTAA				
oct4	GTATTCAGCCAAACGACCATC	487	60	NM_203289.4	Human
	GGAAAGGGACCGAGGAGTACA				
rex1	GGCCGACTACCTCAGACAGATCAT	271	58	NM_020695.3	Human
	GTTCTCTGGCCGCCAGCTCAT				
beta-actin	AGCGAGCATCCCCCAAAGTT	285	53	NM_001101.3	Human
	GGGCACGAAGGCTCATCATT				
bcl-2	GGTCATGTGTGTGGGGAGCGTC	238	60	NM_016993.1	Rat
	TGCACCCAGAGTGATGCAGGCCC				
caspase-3	TCTGACTGGAAAGCCGAAACT	207	60	NM_012922.2	Rat
	AGTGACTGGATGAACCATGAC				
hgf	TGTGTCTGAAGCACCCACC	285	55	NM_000601.4	Human
	GAAATCTTTATCATCCAGCAAAT				
igf-1	AGCACTCACTGACTCTTCTATG	285	60	NM_000618.3	Human
	GGAAGTTTTTGCCTTTCAACTGG				
gapdh	GCAAGTTCAACGGCACAG	141	60	NM_017008.3	Rat
	CGCCAGTAGACTCCACGAC				
bcl-2	GATTGATGGGATCGTTGCCTTAT	173	60	NM_000633.2	Human
	ATTCCAATTCCTTTCGGATCTTT				
caspase-3	TGGAAATGTTCTAAAGGTGGTGAG	293	60	NM_004346.3	Human
	CAAGAAATCTCCCGTGAAATGTC				

### Immunocytochemistry

Human eyelid adipose-derived stem cells cultured in 24-well chamber slides were washed with PBS. After endogenous peroxidase activity was inactivated by 3% hydrogen peroxide, the slides were incubated in a blocking solution, consisting of 1% bovine serum albumin (BSA; Gibco) for 1 hr at room temperature. Between each steps, cells were washed with PBS three times. The cells were then incubated with primary antibodies (Table 1) overnight at 4°C. After being labelled with primary antibody, the cells were then incubated with FITC-conjugated goat antimouse or anti-rabbit IgG (BD Pharmingen) respectively for 2 hrs. Immunoreactive cells were visualized by confocal microscopy (LSM-510; Zeiss Inc, Oberkochen, Germany).

### Neural differentiation of hEASCs

For neural induction, hEASCs were exposed to a cocktail of serum-free medium, as described previously by Hermann *et al*. 30. The cells were treated with Neurobasal medium (Gibco) containing 20 ng/ml human epidermal growth factor (hEGF), 20 ng/ml basic-fibroblast growth factor (bFGF; Millipore Inc, Temecula, CA, USA), 2% B27 (Gibco), 1% penicillin–streptomycin. After 7–9 days, neurosphere-like structures appeared and were subsequently cultured for two to four passages. The medium was changed once per week, and growth factors were added twice per week.

### hEASCs treatment with E2/H_2_O_2_ *in vitro*

To test the effect of E2 alone on hEASCs, cells were incubated with E2 for 24 or 48 hrs before performing cell viability test. Human eyelid adipose-derived stem cells cultures were exposed to varying concentrations of E2 in the culture media for 24 or 48 hrs and cell viability was calculated using the CCK8 assay. The doses of E2 (10^−4^–10^−9^ M) that induce toxicity in hEASCs are likely well above normal physiological levels (10^−12^–10^−9^ M).

To evaluate the potential cytoprotective effect of E2 against oxidative stress-induced hEASCs death, hEASCs were pre-incubated with E2 at various concentrations for 2 hrs followed by by co-treatment of E2 and H_2_O_2_ at a final concentration of 450 μM for 24 hrs. Human eyelid adipose-derived stem cells viability was estimated using CCK8 assay. H_2_O_2_ was freshly diluted from 30% H_2_O_2_ stock solution with DMEM medium to a 450 μM final concentration prior to each experiment.

### Cell labelling and detection

Before implantation, the hEASCs were incubated with 10 μM carboxyfluorescein diacetate-succinimidyl ester (CFDA-SE; Invitrogen) for 15 min., and then washed three times with PBS. To evaluate the survival of implanted hAESCs within the lesions, a non-invasive Kodak FX small animal imaging system Colour CCD tracking system was used to image the implantation sites at 6 weeks after surgery 31.

### Spinal cord injury model

All animal procedures complied with the Guidelines for the Care and Use of Laboratory Animals and were under the supervision of the Institutional Animal Care and Use Committee of Zhejiang University (permit no. zju2010102014). Male SD rats (200–250 g) were purchased from the animal centre of Zhejiang University. Briefly, male SD rats were anaesthetized with 10% chloralic hydras (400 mg/kg bodyweight). A longitudinal incision was made on the midline of the back, exposing the paravertebral muscles. These muscles were dissected away, and clamps were used to immobilize the spinal column. Thoracic vertebra (T9-T10) dorsal laminectomy was performed *via* fine rongeurs. After exposing the spinal cord, a right hemisection was created at T10 by a fine microdissection scissors, which was cut a second time to ensure the lesion was completed according to previous studies 32,33. The cord was then covered with a piece of gelatin sponge (Jinling Inc, Nanjing, China), and the muscles were sutured and the skin closed. The rats were singly housed in a temperature-controlled room at 27°C. Food and water were provided to the rat *ad libitum*. The animals' bladders were manually voided twice a day until the rats were able to regain normal bladder function. A total of 48 adult male Sprague–Dawley rats (200–250 g) were operated in this study.

### Transplantation of hEASCs into SCI rats

The SCI rats were randomly divided into three groups, with 16 animals in each group. The first groups underwent sterile PBS injection. The second groups were injected with hEASCs only. The third groups were treated with both E2 intramuscular injection and hEASCs transplantation. All the three groups received cyclophosphamide (50 mg/kg/day, s.c.; Ruiheng Inc, Jiang Su, China) 1 day prior to transplantation, followed by daily administration until the animals were culled. E2 was pre-administered (100 μg/kg) 15 min. after SCI (7 days before cell transplantation), followed with daily dosing for the next 15 days. Cell transplantation or PBS (control) administration occurred 7 days after SCI.

Transplantation procedures were modified as previously described 34,35. Briefly, the animals were anaesthetized as described previously, the laminectomy site was re-exposed, and scar tissue was removed from the wound site. After immobilization of the spinal process of the lesion site, 5 μl cell suspensions were injected at a rate of 1 μl/min., at a depth of 1.2 mm into the dorsal spinal cord, and at 2 mm rostrally and 2 mm caudally to the injury site with a 10 μl Hamilton syringe (Hamilton Inc, Bonaduz, GR, Switzerland) filled with a total volume of 10 μl (10^6^ cells suspended in PBS). The injury stumps were filled with fibrin glue (PuJi Inc, Hangzhou, China) to stop bleeding in all experimental animals. Control animals received an equal volume of PBS 15 min. after SCI. The needle was removed after 5 min. The rat's muscles were then sutured and the skin was closed.

### Histology and immunofluorescence

Six weeks after SCI, the rats of each group were perfused with isotonic physiological saline followed with 4% paraformaldehyde (Hushi Inc, shanghai, China) in 0.1 M PBS (pH 7.4) under 10% chloral hydrate anaesthesia. Following perfusion, spinal cords were carefully dissected, post-fixed overnight in 4% paraformaldehyde and dehydrated overnight at 4°C in 30% sucrose. The spinal cord was divided into blocks that extended 4 mm cranial and caudal to the injury epicentre respectively, and embedded in OCT and cryosectioned. Cryostat sections (18 μm) were used for haematoxylin and eosin staining and immunofluorescent staining following standard protocols. In addition, a piece of spinal cord of 2-mm-length cranial to lesion was embedded in resin and cut at 1 μm for toluidine blue staining according to standard protocols 36.

Immunofluorescent staining was performed to detect the protein expression of hEASCs *in vivo*. Primary antibodies including anti-glial fibrillary acidic protein (GFAP; 1:400; Chemicon Inc., Temecula, CA, USA), anti-MAP2 (1:500; Chemicon Inc.,), anti-human nuclei (1:200; Chemicon Inc.,) and anti-NF200 (1:200; Chemicon Inc.,) were incubated overnight at 4°C. Goat anti-rabbit (Alexa Fluor 546) or goat antimouse (Cy3) secondary antibodies were used at a dilution of 1:500 in 1% BSA in PBS. Following 2 hrs of incubation, all sections were washed in PBS. Sections without primary antibody incubation were processed in the same manner as negative controls. All the histology slides were viewed and digitally photographed by a BX41Olympus microscope (Olympus Inc., Tokyo, Japan). Immunofluorescence was visualized by confocal microscopy (LSM-510; Zeiss Inc).

### Behavioural assessments

The BBB (Basso, Beattie and Bresnahan) locomotor rating scale was adopted to evaluate the functional recovery of hindlimbs during open field locomotion 37,38. For testing, rats were placed in an open field (80 × 130 × 30 cm) with a pasteboard covered non-slippery floor for 4 min. In each testing session, the animals were observed individually by two examiners who were blinded to the animals' treatment, and the assay was conducted once per week until 6 weeks after transplantation. The BBB is a 21-point scale designed to assess hind limb locomotor recovery after injury to the thoracic spinal cord.

The grid-walk test has been validated as an assessment of sensorimotor function 38,39. Paw placements of hind limbs was tested as animals walked on an elevated plastic coated wire mesh grid (40 × 45 cm with 2 cm^2^ grid spaces). Animals were placed on the grid for 3 min. and allowed to walk freely across the platform. A misstep occurred when the entire foot fell through the grid. Injured limb was scored for the total number of steps and the percentage of missteps. The grid-walk test was carried out once per week on the experimental animals.

### TUNEL staining

To determine whether spinal cord damage was associated with cell death by apoptosis, we performed terminal deoxynucleotidyl transferase-mediated digoxigenin-dUTP-biotin nick-end labeling (TUNEL) assay in the peri-lesional spinal cord tissue. Frozen sections prepared for immuno-histochemistry as described previously, were assayed with a TUNEL apoptosis detection kit (Roche Diagnostics Inc, Laval, QC, Switzerland) and nuclear staining with haematoxylin according to the manufacturer's instruction. Briefly, frozen sections of three groups (*n* = 4) were perfusion-fixed with 4% paraformaldehyde (pH 7.4). Endogenous peroxidase was inactivated by 0.3% H_2_O_2_ in methanol (v/v) for 15 min. at room temperature and then washed with PBS. For cell permeabilization, slides were immersed in 0.1% Triton X-100 in 0.1% sodium citrate (v/v) for 2 min. on ice, and then rinsed twice in PBS for 5 min. Tissue sections were incubated in a humid chamber for 60 min. with the reaction mix. POD-conjugated avidin was applied to the sample, and then incubated at 37°C for 30 min. Positive-stained POD-labelled cells were visualized with diaminobenzidine substrate. Positive control sections were incubated with 200 U/ml DNase 1, grade 1 (Roche Diagnostics) in 50 mM Tris-HCl (pH 7.5) to induce DNA strand breaks prior to labelling procedures. All photographs were taken with a microscope (Olympus Inc.). Quantification of TUNEL-positive cells was accomplished by counting the number of cells labelled positively under a 40× objective.

### Reverse transcription polymerase chain reaction (RT-PCR) and quantitative real-time PCR analysis

To evaluate the combined effect of hEASCs and E2, *in vivo* expression of selected genes of interest (GOIs) (Table 2) was assessed by quantitative PCR with Brilliant SYBR Green QPCR Master Mix (TakaRa Bio Inc., Shiga, Japan) and a Light Cycler apparatus (ABI 7900HT; Applied Biosystems Inc., Carlsbad, CA, USA). RNA isolation was processed 1 week after cell transplantation. Approximately 200 mg of spinal cord tissue around the injury site was prepared for total RNA isolation according to the manufacturer's instructions. cDNA was reverse transcribed from the extracted mRNA, as previously described. To avoid interspecies cross-reactivity of the primer pairs between human and rat genes, human-specific primers were designed. The relative level of expression of each target gene was then calculated as −2^ΔΔCt^. Each sample was replicated at least thrice.

### Statistical analysis

Student's *t*-test was used for comparison between two experimental groups. One-way anova was used to compare the three experimental groups, together with the Student–Newman–Keuls test and Tukey HSD for pairwise inter-comparison of the subgroups. Data were expressed as mean ± SD. *P* < 0.05 was considered statistically significant.

## Results

### Characterization of human eyelid adipose-derived stem cells

**Fig 1:**
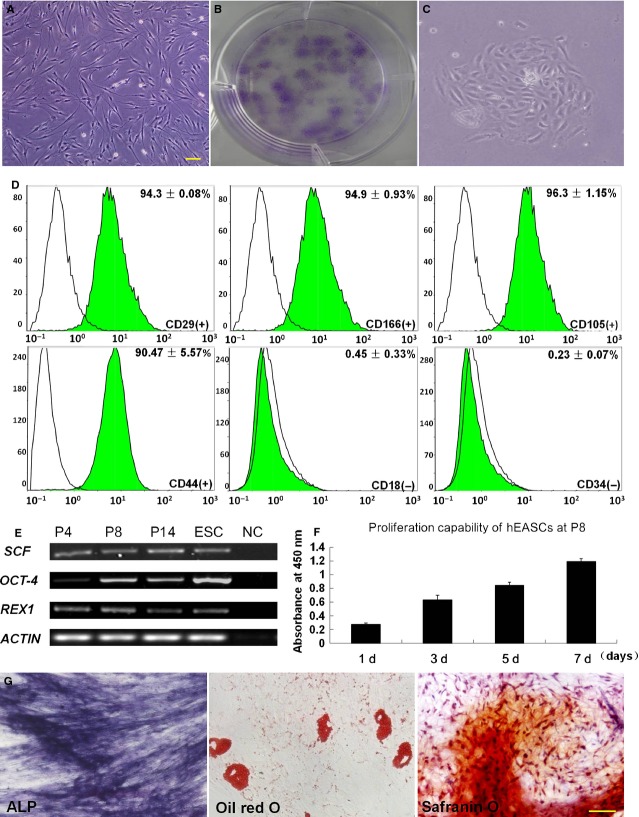
Isolation and characterization of human eyelid adipose-derived stem cells (hEASCs). (A) Morphology of hEASCs at p3. (B) Crystal violet staining to visualize colonies formed by hEASCs. (C) Morphology of a colony formed by hEASCs. (D) Flow cytometry analysis of hEASCs. hEASCs at p4 were strongly positive for mesenchymal stem cells surface markers CD44, CD29, CD166, CD105, and negative for haematopoietic makers CD18, CD34. (E) Stem cell-specific gene expression of by hEASCs at p4, p8, p14 and hESCs. (F) Proliferation capability of hEASCs at P8. (G) Differentiation potential of hEASCs at p5 into osteoblasts, adipocytes and chondroblasts. ALP staining showing osteogenic differentiation of hEASCs; Oil red O staining showing adipogenic differentiation of hEASCs; Chondrogenic differentiation of hEASCs revealed by safranin O staining. Scale bars = 200 μm (A), and 50 μm (G). Abbreviations: hEASC, human eyelid adipose-derived stem cell; hESC, human embryonic stem cell; NC, negative control; P, passage.

After 3-weeks culture of cells isolated from human eyelid adipose tissues, plastic-adherent cells with unique morphology were obtained and are referred to as hEASCs. These cells exhibited bipolar morphology (Fig. 1A). Culture of hEASCs was established after subsequent expansion and grew for 14–15 passages before reaching senescence. To determine the clonogenicity of hEASCs, the cells were seeded at very low seeding densities (100 cells were seeded in the 6 cm dish, three cells/cm^2^). After 10–12 days, monoclonal colonies (Fig. 1C) were detected by methyl violet staining (Fig. 1B). The clonal formation ability of hEASCs at passage 3 was ∼20 colonies/100 cells. To phenotypically characterize the isolated colonies, FACS was performed with various cell surface markers including MSCs-specific markers and haematopoietic stem cell markers (Table 1). FACS analyses revealed that the hEASCs were positive for surface antigens such as CD44 (90.47 ± 5.57%), CD29 (94.3 ± 0.08%), CD166 (94.9 ± 0.93%) and CD105 (96.3 ± 1.15%), but negative for CD18 (0.45 ± 0.33%) and haematopoietic markers CD34 (0.23 ± 0.07%; Fig. 1D). Throughout the culture, RT-PCR analysis showed that hEASCs at passages 4, 8 and 14 expressed stem cell-specific genes such as *OCT4*, *REX1* and *SCF*. Furthermore, hEASCs possessed good proliferative capacity *in vitro* (Fig. 1E and F). Taken together, these results suggested that hEASCs possessed stem cell properties. To evaluate the multipotent differentiation potential of hEASCs, the cells were cultured in adipogenic, osteogenic and chondrogenic differentiation-inducing media. Our results indicated that hEASCs were able to differentiate into the osteogenic, adipogenic and chondrogenic lineages as shown by respective ALP, Oil red O and safranin O staining respectively (Fig. 1G). These results thus demonstrated that hEASCs had the potential to differentiate into various mesodermal lineages.

**Fig 2:**
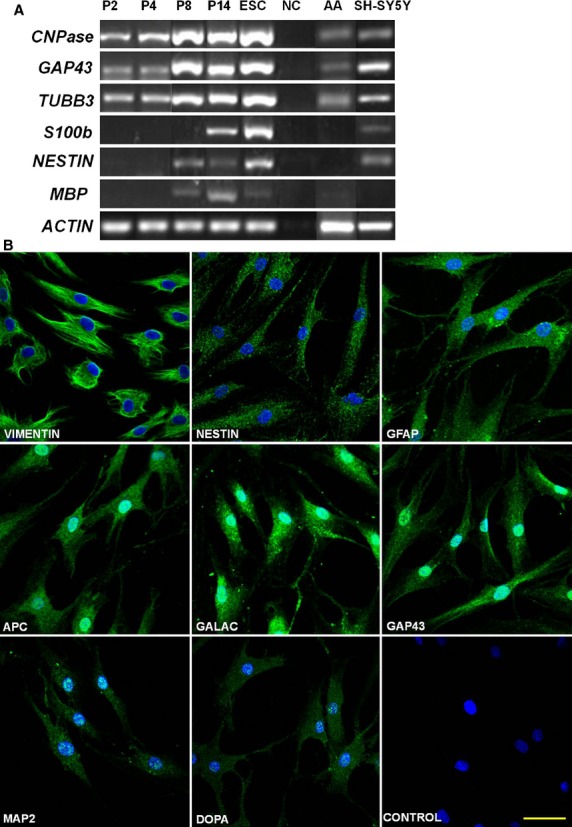
Neural properties of human eyelid adipose-derived stem cells (hEASCs). (A) Neural-related genes expression of hEASCs at p2, p4, p8 and p14. (B) Neural-related proteins immunocytochemistry of hEASCs at p8. Neural-related proteins showed green colour, and blue is the DAPI staining. Scale bar = 50 μm. Abbreviations: hEASC, human eyelid adipose-derived stem cell; hESC, human embryonic stem cell; NC, negative control; p, passage; AA, human abdominal adipose-derived stem cells at p3; NB, neuroblastoma cells.

A previous study has shown that hEASCs originated from the neural crest.^8^ Consistent with this observation, our RT-PCR data also showed that hEASCs expressed various neural lineage genes including ectoderm-or neural progenitor-specific genes such as *NESTIN,* neuronal genes *TUBB3*, *GAP43,* and *MBP*, glial gene *CNPase*, and astrocyte gene *S100b* at different passages (Fig. 2A). Human neuroblastoma cell line (SH-SY5Y) showed distinct expression of all of these genes except *MBP*. In contrast, human abdominal adipose-derived MSCs at passage 4 did not significantly express all of the above genes except *CNPase*, *TUBB3* and *GAP43*. Immunocytochemical analyses further confirmed that hEASCs at passage 8 spontaneously expressed a variety of neural-related markers including Nestin, GFAP, APC, MAP2, galactocerebroside (Galac), DOPA and GAP43, in addition to the mesoderm/endoderm-specific marker Vimentin (Fig. 2B). However, the mature neuronal marker NeuN was not expressed (data not shown). These results suggested that unlike MSCs, hEASCs retained neural characteristics even after long-term culture *in vitro*.

The expression of many neural lineage-associated genes by hEASCs prompted us to further evaluate their ability to differentiate into neural lineages. In the presence of hEGF and bFGF, the majority of the neural-induced hEASCs (NI-hEASCs) developed distinct bipolar or multipolar morphology with branched processes and increased refraction compared with the control (Fig. S1A and B). After 9 days of induction, neural stem cell like-floating neurospheres were observed (Fig. S1C and D). Nestin, MAP2, APC and GFAP expression increased following neuronal induction (Fig. S1E and L). Staining was evident in both the cytoplasm and nuclei of the majority of cells. Optical density analysis of immunocytochemical staining before and after neuroinduction showed that the change in MAP2 expression level was significantly different (Fig. S1M). These results demonstrated that hEASCs possessed the capacity to differentiate into neural stem-like cells, thus holding promise for neural differentiation.

### E2 protects hEASCs against H_2_O_2_-induced cytotoxicity *in vitro*

**Fig 3:**
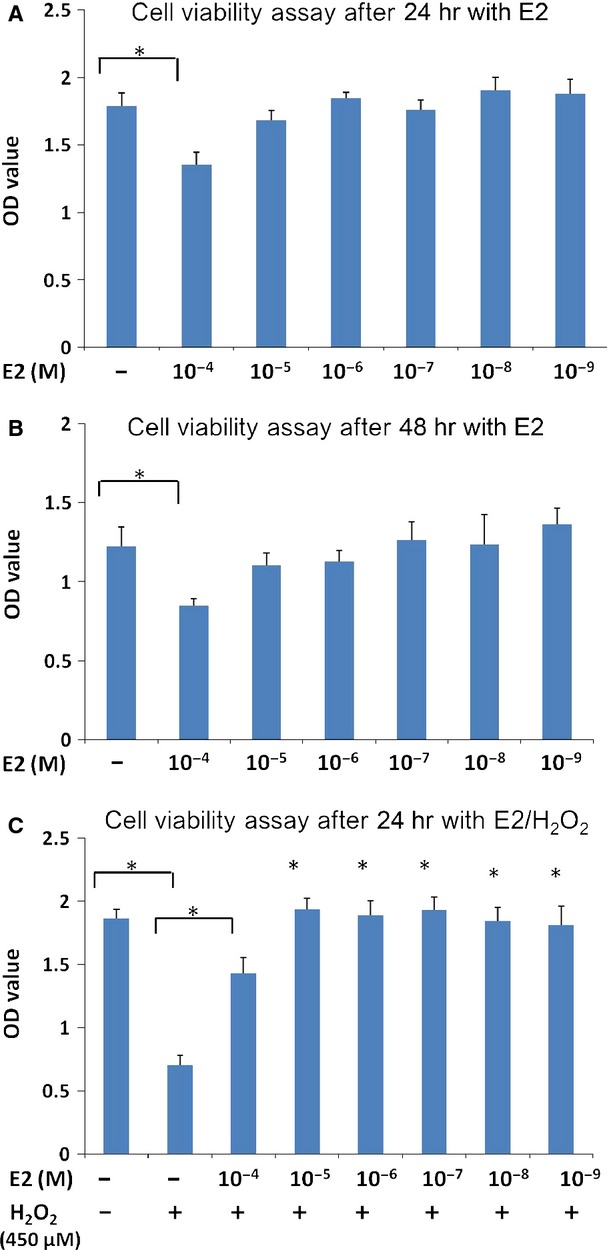
The effect of E2 on hEASCs/hydrogen peroxide (H_2_O_2_). (A and B) Toxic effect of E2 on hEASCs. High concentrations of E2 (10^−4^ M) significantly decreased the survival of hEASCs (*P < 0.05 as compared with cells administered no E2). There were no significant differences in cell survival at the concentration of E2 (10^−9^–10^−5^ M) treatment at the 24 and 48 hrs time-point. (C) Dose response effects of E2 on H_2_O_2_-induced hEASCs death. Exposure to H_2_O_2_ in untreated hEASCs caused a significant reduction in cell survival compared with the control cells (indicated by *P < 0.05). Administration of E2 at concentrations between 10^−4^ and 10^−9^ M significantly increased the percentage of hEASCs survival as compared with cells treated with H_2_O_2_ alone. The OD value is shown as mean ± SD (*P < 0.05 as compared with H_2_O_2_ treatment alone). *n* = 8, from three separate cultures.

The effect of E2 on hEASCs survival was measured after either a 24 or 48 hrs incubation time (Fig. 3). In this experiment, hEASC cultures were exposed to varying concentrations of E2 in the culture media for 24 or 48 hrs and cell viability was calculated using the CCK8 assay. We found that incubation with E2 in a dose range from 10^−9^ to 10^−5^ M for 24 hrs did not induce significant changes in cell survival as compared with the control groups. However, the higher doses of E2 (10^−4^ M) caused a significant reduction in cell survival as compared with the control groups after either a 24 or 48 hrs. The doses of E2 that induce toxicity in hEASCs are likely well above normal physiological levels (10^−12^–10^−9^ M).

To evaluate the potential cytoprotective effect of E2 against oxidative stress-induced hEASCs death, hEASCs were treated with E2 at various concentrations for 2 hrs followed by exposure to H_2_O_2_ at a final concentration of 450 μM for 24 hrs. Human eyelid adipose-derived stem cells viability was estimated by CCK8 assay. When treated with H_2_O_2_ for 24 hrs, hEASCs viability was significantly decreased as compared with control. Incubation with E2 at concentrations between 10^−9^ and 10^−4^ M 2 hrs prior to H_2_O_2_ treatment significantly increased the OD value as compared with H_2_O_2_ treatment alone (Fig. 3). These data suggest that E2 in the low micromolar range is protective against oxidative stress in hEASCs.

### Experimental schematics and fluorescence tracking of grafted hEASCs in the SCI model

**Fig 4:**
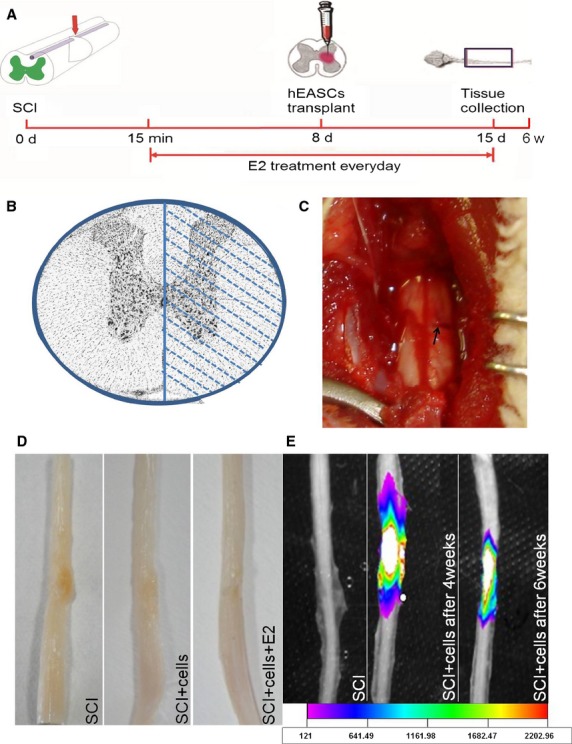
Transplantation of hEASCs and E2 administration for spinal cord injury treatment. (A) Diagram of the experimental timeline detailing. (B–C) Diagram showing right unilateral hemisection of an adult spinal cord. Complete lesion was made on one side of the spinal cord at 8th thoracic segments (T8). (D) General morphology of spinal cord after 6 weeks. (E) A positive orange fluorescence signal in the repair sites were observed by Colour CCD tracking at cell groups. The colour coding and grades showed the intensity of fluorescence signal.

The experimental schematics to evaluate our hypothesis is presented in Figure 4A. The diagram showed right unilateral hemisection of an adult spinal cord (Fig. 4B). A complete lesion was made on the right side of the spinal cord. After 6 weeks, the spinal cords were explanted from rats (Fig. 4C and D). Positive orange fluorescence signals at the repair sites were examined by Colour CCD analysis, indicating the survival of implanted CFDA-SE-Labelled hEASCs at 4 and 6 weeks after surgery (Fig. 4E). These findings indicated that hEASCs could survive for a substantially long duration *in vivo*.

### Survival and differentiation of the transplanted hEASCs

**Fig 5:**
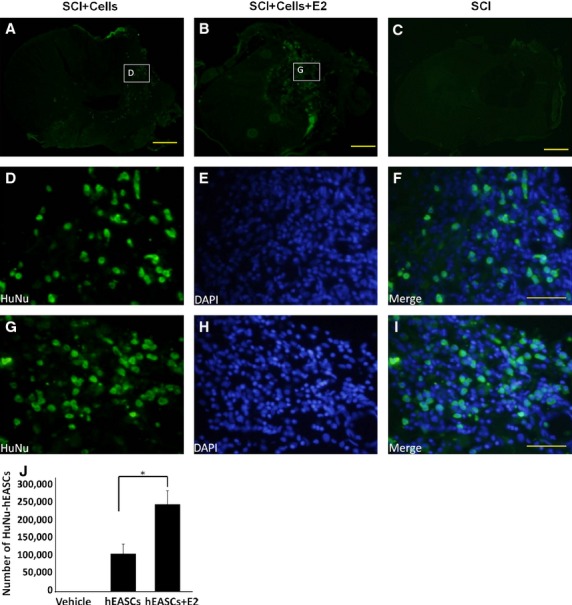
Survival of grafted hEASCs after 6 weeks *in vivo*. (A–C) The distribution of hEASCs was detected using anti-human-positive nuclei (anti-HuNu) staining in the hEASCs groups (A), E2 +  hEASCs groups (B) and spinal cord injury alone groups (C) respectively. (D–I) A higher magnification micrograph from the box in (A and B) respectively. Green colour is the anti-human-positive nuclei (anti-HuNu) staining. Blue is the DAPI staining. (J) Quantification of hEASCs survival. *P < 0.05 as compared with hEASCs transplanted alone groups. Scale bar = 500 μm (A–C), 50 μm (D–I).

To further determine whether any of the transplanted cells survived, we performed nuclear immunocytochemistry studies to detect human-specific antigens (anti-HuNu; Fig. 5). The presence of hEASCs was detected by anti-HuNu in all transplanted animals, thus confirming xenograft survival. As expected, control animals did not exhibit such labelling (data not shown). Transplanted hEASCs survived and localized to the injury site during the 6-week study period. At the injury epicentre, anti-human positive cells were identified throughout the transverse plane, and were concentrated around the area of former cavitation (Fig. 5A and B). Higher magnification images from the inset box in (A-B) were respectively displayed as Fig. 5C–H. Rodent cells were also present within this area, and were identified by 4′,6-diamidino-2-phenylindole (DAPI) staining. To quantify the number of anti-HuNu-labelled hEASCs in the three groups, unbiased stereology was conducted on serial sections throughout the lesion epicentre of the experimental animals. The number of surviving labelled hEASCs was 108,333 ± 25,658 in the hEASCs alone groups. Significantly more labelled hEASCs (242,000 ± 38,301) were counted in the E2-hEASCs groups (*P* < 0.05; Fig. 5I). These data indicated that post-SCI administration of E2 caused a nearly twofold increase in survival of transplanted hEASCs, and support the hypothesis that E2 protects hEASCs.

The fate of surviving hEASCs after implantation was examined by double-labelling for individual glial/neural markers (GFAP, GALAC and MAP2) with anti-HuNu staining. Cells co-labelled with-HuNu and mature neuronal marker MAP2 antibodies (red)/the mature oligodendrocyte marker GALAC (red) were detected within the white matter region (Fig. S2A–F), but no cells co-labelled with anti-HuNu and GFAP antibodies were observed at 6 weeks after transplantation (Fig. S2G–I). These results showed that hEASCs have the potential to differentiate into neurons and oligodendrocytes, but not astrocytes. However, the differentiation ratio is very low.

### hEASCs transplantation reduced cavity formation *in vivo*

**Fig 6:**
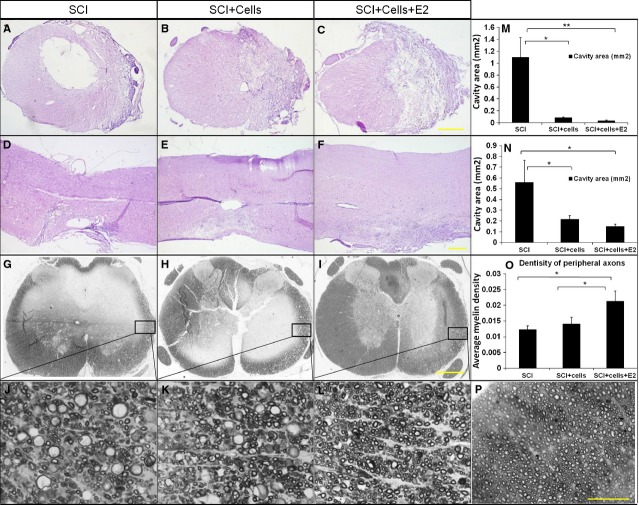
Histopathology following spinal cord injury (SCI). Transverse (A–C) and longitudinal (D–F) haematoxylin and eosin-stained section at the injury epicentre depicts the loss of gray matter and white matter, respectively, 6 weeks post-operatively in different groups. (G–I) Low magnification imaging reveals pathology 2 mm rostral to the injury epicentre of three groups by Toluidine blue staining. (J–L) Higher magnifications of the closed rectangle regions in G–I respectively, illustrated differences in peripheral myelinated axon density. (P) Higher magnifications of normal myelin structure in uninjured spinal cord. (M and N) The cavity areas were significantly decreased in E2-hEASCs groups *versus* the hEASCs groups and the SCI groups in transverse sections (M) and longitudinal section (N; *P < 0.05) respectively. (O) Average myelin denstity of peripherally myelinated axons 2 mm rostral to the injury epicentre (*P < 0.05, **P < 0.01) Scale bar = 500 μm (A–I), 50 μm (J–L and P). Data represent mean ± SD, *n* = 3, in each group.

To investigate the effect of hEASCs transplantation on the reduction of cavity formation *in vivo*, we performed haematoxylin and eosin staining on the transverse and longitudinal sections of the differentially treated spinal cords after SCI. We found that the injury epicentre in the spinal cords from the PBS control groups developed maximal cavum with widespread loss of white matter and gray matter at 6 weeks after SCI. By contrast, there was significantly reduced size of cavum after cell transplantation in both the hEASC groups and E2-hEASC groups (Fig. 6A and B). This observation was confirmed by quantitative estimation from the longitudinal and transverse sections of the injury centre (Fig. 6E and F). The mean cavity dimensions of the PBS groups were 0.56 ± 0.2 mm^2^ and 1.1 ± 0.33 mm^2^ at the longitudinal and transverse sections respectively. Notably, a significant decrease was observed in the hEASC groups, with the corresponding dimensions of the cavity being 0.21 ± 0.03 mm^2^ for the longitudinal section (*P* < 0.05 *versus* PBS groups) and 0.85 ± 0.01 mm^2^ for the transverse section (*P* < 0.05 *versus* PBS groups) respectively. In the combination groups, the corresponding dimensions of the cavity were 0.15 ± 0.02 mm^2^ longitudinally (*F* = 10.12, *P* < 0.05 *versus* PBS groups) and 0.03 ± 0.01 mm^2^ transversely (*F* = 29.11, *P* < 0.01 *versus* PBS groups). These results thus demonstrated that hEASCs transplantation could significantly reduce cavity formation after SCI.

### E2 administration reduced demyelination *in vivo*

Toluidine blue staining was used to determine the number and shape of myelinated axons after treatment of SCI. Histological examination indicated that myelin pathology 2 mm rostral to the injury epicentre exhibited scattered demyelination in the PBS groups and hEASC groups, which were improved after E2 administration (Fig. 6C). Higher magnification micrograph from the inset box in (C) showed the morphology of the myelin sheath (Fig. 6D). Compared with the PBS groups and hEASC groups, the E2-hEASC groups displayed higher numbers of myelin sheath (*F* = 21.042,*P* < 0.01 *versus* PBS groups & hEASC groups) (Fig. 6G). The results suggested that E2 administration could protect the myelin sheath, thus reducing demyelination.

### E2-hEASC groups displayed better recovery of motor function after SCI

**Fig 7:**
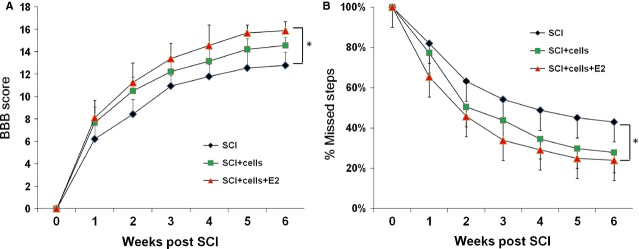
E2-hEASCs combination improves hindlimb motor functional recovery following spinal cord injury (SCI). (A) Weekly BBB (Basso, Beattie and Bresnahan) score 1–6 weeks after transplantation. (B) Weekly Grid walking score 1–6 weeks after transplantation. The BBB scores of the hEASCs groups and the SCI groups were not significantly different at 6 weeks after SCI. By contrast, the E2-hEASCs groups was significantly different from the SCI groups (repeated measures anova post hoc Tukey test, *F* = 25.4,*P < 0.05 *versus* PBS groups) but not significantly different with the hEASCs groups. The gait pattern characterization results of Grid walking were consistent with the BBB score. The percentage of missteps of hind paws was dramatically reduced in E2-hEASCs groups (repeated measures anova post hoc Tukey test, *F* = 7.302, *P* < 0.05 *versus* PBS groups). Data represent mean ± SD, *n* = 12 per group.

Behavioural performance was evaluated using the BBB locomotor rating scale and Grid-walking test at weekly intervals for up to 6 weeks. Although all rats exhibited a gradual improvement over time, the BBB scores of the hEASC groups and the PBS groups were not significantly different, with corresponding values of 14.55 ± 0.72 and 12.77 ± 1.20 respectively at 6 weeks after transplantation. By contrast, the E2-hEASC-treated rats showed better outcome than other two groups, with a final score of 15.88 ± 0.78 (Fig. 7A), which was significantly different from the PBS groups (*P* < 0.05, *versus* PBS groups, repeated measures anova post hoc Tukey test, *F* = 25.4) but not significantly different with respect to the hEASC groups. The gait pattern characterization results were consistent with the BBB score. All the PBS controls exhibited hindlimb dysfunction with features of sweeping or plantar placement with no weight support, whereas some of the animals in the hEASC-treated groups displayed occasional forelimb and hindlimb coordination, but there was no significant difference between these two groups. However in the E2-hEASC groups, the percentage of missteps of hind paws was dramatically reduced (Fig. 7B; repeated measures anova post hoc Tukey test, *F* = 7.302, *P* < 0.05 *versus* PBS groups). Taken together, these results indicated that hEASCs treatment alone had little efficacy after SCI. Pre-administration E2 and hEASCs could improve the functional recovery of paralyzed rats in the rat SCI model.

### E2 administration reduced cellular apoptosis, promoted the survival of grafted hEASCs and increased the expression of growth factors by hEASCs *in vivo*

**Fig 8:**
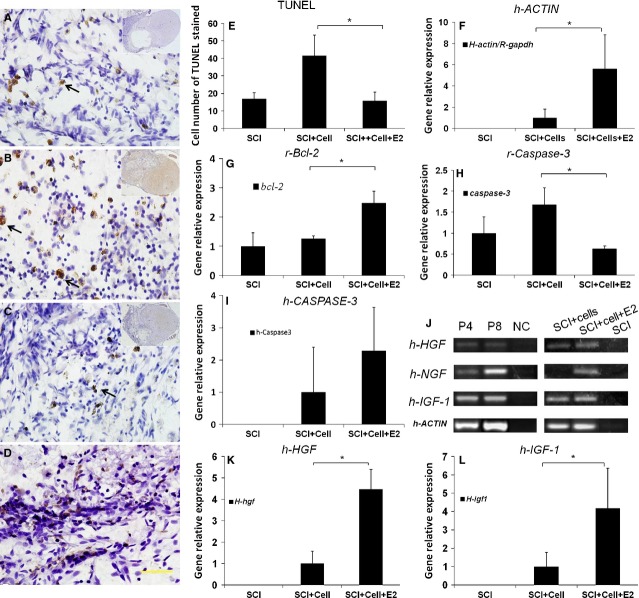
E2 anti-apoptotic and promote growth factors expression *in vivo*. (A–C) Representative micrographs of TUNEL-positive cells counterstained with haematoxylin and eosin in spinal cord injury (SCI) groups (A), hEASCs groups (B), and E2-hEASCs groups (C) respectively, as the black arrow shown. (D) TUNEL-positive cells in positive sample. (E) Cell number quantification of TUNEL-positive cells. *P < 0.05 *versus* PBS groups. (F–H) Rat-bcl-2 specific activity was significantly increased (F), while rat caspase-3 was decreasd (H) in E2-hEASCs-groups. (I) Humancaspase-3 activity has no significant difference between cell-grafted groups. (J) Human gene expression analyses of HGF, NGF, IGF-1, β-ACTIN expression *in vitro* (p4, p8) and *in vivo*. (G, K and L) Real-time PCR analyses of β-ACTIN (G), h-HGF (K), h-IGF-1 (L) mRNA expression in three groups after SCI Note that E2 treatment significantly increased h-HGF, h-IGF-1 and β-ACTIN mRNA expression after SCI as compared to that of alone cells groups. *P < 0.05 *versus* hEASCs groups. Data represent mean ± SD (*n* = 3). Scale bar = 50 μm (D).

Spinal cord sections of the rostra region from each group were analysed for TUNEL-positive cells (Fig. 8A–C). As a prelude to investigations on apoptotic gene activation, the occurrence of apoptosis in the spinal cord after SCI was analysed using a cell apoptosis detection kit (TUNEL) and nuclear staining with haematoxylin. The presence of apoptotic cells was identified in the injured spinal cord. We used a positive sample as the positive control (Fig. 8D). Quantitative estimation of apoptotic cells indicated that PBS control rats had an average of 16 ± 3 apoptotic cells per section at 2 mm rostral to T9. In hEASC groups, the number of apoptotic cells increased up to 41 ± 11, within the dorsal white matter, especially around the lesion epicentre. Notably, we observed significant reduction in the number of apoptotic cells (15 ± 4) in the E2-hEASC groups (Fig. 8E; *F* = 39.731, *P* < 0.01 *versus* PBS groups).

Real-time PCR further confirmed apoptotic gene expression. The results showed that expression of the rat anti-apoptotic genes *Bcl-2* was significantly increased after E2 treatment compared with the hEASC groups (Fig. 8G; *F* = 5.527, *P* < 0.05 *versus* PBS groups). Rat *Caspase-3* activity increased in the hEASC groups, whereas it was significantly decreased in the E2-hEASC groups (Fig. 8H; *F* = 6.347, *P* < 0.05 *versus* hEASC groups). We also analysed the gene expression levels of human *Caspase-3* and *Bcl-2 in vivo*. Real-time PCR results showed that human *Caspase-3* activity displayed no significant difference in the hEASC groups with or without E2 administration (Fig. 8I). Human *Bcl-2* activity was not detected in both groups. These results suggested that E2 improved the local injury environment and protected cells from apoptosis mainly through modulation of host (rat) genes, rather than exerting a direct effect on the grafted cells. To determine whether pre-administration of E2 after SCI *in vivo* could enhance the survival of transplanted hEASCs, we examined the expression of human β*-actin* by *RT-PCR* after SCI (7 days after transplantation), *rat gapdh* as the endogenous control. Although this method is not very serious, it reflected the human gene expression quantity in rat model to some extent. The gene expression level in E2-hEASC groups was five times higher than that in hEASC groups (*P* < 0.05; Fig. 8F). These data thus indicated that pre-administration of E2 promoted the survival of hEASCs after transplantation through modulating the expression of rat apoptosis-related genes (Caspase-3/Bcl-2).

On the other hand, RT-PCR analyses of mRNA expression *in vitro* (p4, p8) and *in vivo* indicated that the hEASCs had the capacity to express *HGF, NGF* and *IGF-1* (Fig. 8J), and the expression levels were significantly improved after E2 administration (Fig. 8K and L). These results indicated that hEASCs might secrete some growth factors to improve the local injury environment and support the histological and functional restoration of SCI at 6-weeks after transplantation. Furthermore, E2 administration could augment the beneficial effects of the transplanted cells.

## Discussion

This study provided the first direct experimental evidence of the protective effects of E2 against H_2_O_2_ insult in hEASCs *in vitro*, which are similar to the role of E2 in several other cell types from others studies 15,20. After SCI, reactive oxygen species (ROS) was thought to be one of the major events contributing to secondary injury 1. E2 protects hEASCs against H_2_O_2_-induced damage; this has significant implications with regard to SCI and therapeutic approaches. Intriguingly, we also demonstrate in this study that E2 administration not only enhanced survival of transplanted hEASCs after SCI, but also made the local injury microenvironment more conducive for tissue regeneration by reducing cellular apoptosis. The surviving hEASCs in turn filled up the cavity at the injury site, and secreted various growth factors. Transplantation of hEASCs together with E2 administration synergistically improved motor function recovery by SCI rats. These findings thus provided a new and promising chemo-cell cocktail strategy for SCI treatment.

It was previously reported that hEASCs constituted a unique stem cell niche that possesses the characteristics of both MSCs and neural stem cells 8,9. Within *in vitro* culture, hEASCs displayed some typical characteristics of MSCs such as expression of CD105, CD29, CD166 and CD44. However, consistent with previous studies, hEASCs spontaneously expressed many neural stem cell-related mRNAs and proteins, unlike MSCs derived from abdominal adipose tissues 8. This difference can likely be attributed to the origin of hEASCs from the neural crest, but not trunk adipose-derived MSCs 40,41. In mice, it has been shown that cephalic neural crest cells generate a subset of facial adipocytes, but do not contribute to the truncal adipose cell lineage during normal development 42. Wrage *et al*. also demonstrated that mouse truncal adipose-derived MSCs do not represent a neural crest-derived population residing in adult adipose tissue, as demonstrated by Wnt-1 lineage tracking analysis 43. Although neuroepithelial cells contribute to the earliest wave of MSC differentiation, Takashima *et al*. demonstrated that these MSCs appeared to be replaced by as-yet-unidentified cells during later embryogenesis 44. These studies suggested that hEASCs are neural crest-derived multipotent stem cells that are readily distinguishable from trunk adipose-derived MSCs, and which constitute a distinct niche within eyelid adipose tissue that might contribute to repair of damaged neural tissue. wTherefore, hEASCs, owing to their neural properties, may represent a superior candidate to other cell types as seed cells for transplantation therapy of CNS injuries. Nevertheless, while cell functionality was investigated with electrophysiology, we were not able to achieve depolarization and repolarization, implying that the differentiated cells after induction did not possess the full functional properties of neurons. Marra reported that to date, no research group has ever provided irrefutable evidence that adipose-derived stem cells are capable of differentiating to mature functional neuronal cells *in vitro* 45. Neural induction protocols *in vitro* therefore need to be further optimized in future studies.

E2 protected hEASCs against H_2_O_2_-induced cell death *in vitro*. Incubation with E2 at a certain concentration prior to H_2_O_2_ treatment significantly increased the cell viability. H_2_O_2_ cellular consumption requires the use of high initial doses of H_2_O_2_ (typically 100 μM–1 mM), which may affect the redox homeostasis of cells and cause oxidative stress and negative responses 20. Thus, we chose an approximate middle dose 450 μM H_2_O_2_ as the final concentration of H_2_O_2_. These data suggest that E2 in the low micromolar range is protective against oxidative stress in hEASCs. These results were consistent with the previous studies about the cytoprotetion of E2 15,20,21. However, this is the first evidence that E2 has the protective effect on hEASCs *in vitro*.

In this study, undifferentiated hEASCs successfully engrafted onto the lesion of the SCI rat models after 6 weeks. Post-SCI administration of E2 protects hEASCs and improves their survival ability after transplantation. Furthermore, human growth factors (IGF-1, NGF and HGF*)* can be secreted by the transplanted hEASCs *in vivo*. Stem cell transplantation may also have a protective effect at the injury site by improving the local microenvironment and local homeostatic state 46,47. As previous studies have reported, growth factors secreted by stem cells can prevent neuronal cell apoptosis and improve motor function after acute SCI 48,49. The positive results of our *in vivo* study provide optimism for hEASCs as a potential therapeutic candidate for post-SCI cell transplantation with clinical implications in neurological applications. Nevertheless, we observed massive apoptotic cell death from TUNEL staining in the sole hEASC groups, suggesting that a large number of hEASCs failed to survive following transplantation. High levels of apoptosis of transplanted cells have previously been well-documented 50–52. This thus suggests that transplantation of hEASCs alone may not be sufficient for efficient repair of SCI. The treatment of this multifactorial injury will likely require combination therapy to address disparate injury components 53.

E2 pre-administration has been shown to promote cell survival in the spinal cord after SCI 54,55. In our study, we went a step further to demonstrate that E2 promoted the survival of grafted hEASCs and significantly enhanced the efficacy of hEASC transplantation therapy of SCI. This effect can likely be attributed to reduction of massive cell apoptosis at the acute phase of SCI through modulation of the expression of Caspase-3/Bcl-2, thereby making the microenvironment of the injury site more conducive for healing 51,56. Consequently, the surviving hEASCs may contribute to higher secreted levels of human growth factors, increased remyelination and distinct improvement in hind limb motor function. Sribnick *et al*. also reported that post-injury oestrogen treatment of chronic SCI improved locomotor function in rats 57. The robust myelin sheath protection in this model might also suggest that this protective effect was because of the administered oestrogen. These results were consistent with the studies of Yune and his colleague 54. Additionally, Ritz and Hausmann also demonstrated that E2 could protect the spinal cord by promoting cytokine release 18. Thus, we speculated that pre-E2-hEASCs treatment might suppress cell apoptosis and enhance functional recovery to a certain extent *via* modulation of apoptosis gene expression, as well as through secretion of growth factors into the surrounding tissue.

## Conclusion

It was demonstrated that E2 administration protected the cell survival *in vitro* and *in vivo*, enhanced the therapeutic efficacy of hEASCs transplantation and facilitated motor function recovery after SCI. The efficacy of cell therapy alone for SCI repair was limited, which could be significantly enhanced by E2 administration. These observations clearly demonstrated that a potential therapeutic paradigm might be the combination of the unique properties of hEASCs and E2, which protects the transplanted cells from apoptosis through modulation of apoptosis gene expression, as well as enhance secretion of growth factors by the transplanted cells. These growth factors could further improve the local environment of the lesion, protect the myelin sheath and improve histological and functional outcome. Hence, this novel chemo-cell cocktail treatment strategy for SCI holds much promise for future clinical applications.

## Supporting information

Figure S1 Neuroinduction of hEASCs (A): Morphology of uninduced hEASCs at p8 (B): Morphology of neural-induced hEASCs after 4 days at p8. (C, D): Morphology of hEASCs at p8 after neural-induced 10 days. The spherical floating aggregates were observed. (E–L) Immunocytochemistry of hEASCs before and afer neuroinduction at p8 (GFAP, APC, Nestin, MAP2). (M) Optical density analysis of immunocytochemistry before and after neuroinduction. Scale bar = 50 μm (A), 20 μm (B), 50 μm (E–L). Abbreviations: p, passage.

Figure S2 The fate of surviving hEASCs after 6 weeks *in vivo*. (A–F): Cells co-labelled with anti-HuNu (green) and mature neuronal marker MAP2 antibodies (red)/the mature oligodendrocyte marker GALAC (red) were detected within the white matter region. (G–I): No cells colabelled with anti-HuNu and GFAP antibodies were observed at 6 weeks after transplantation. These results showed that hEASCs have the potential to differentiate into neurons and oligodendrocytes, but not astrocytes. Nuclei are stained by 40,6-diamidino-2-phenylindole (DAPI). Scale bar = 20 μm (A–I)

Figure S3 Anti-Ki67 and -HuNu Immunofluorescence of hEASCs after 6 weeks *in vivo*.
