# Supplementary figures and images for: 17β-Estradiol Protects Human Eyelid-Derived Adipose Stem Cells against Cytotoxicity and Increases Transplanted Cell Survival in Spinal Cord injury
Source: J Cell Mol Med. 2013 Dec 22;18(2):326–43. doi: 10.1111/jcmm.12191 (PMC3930419; doi:10.1111/jcmm.12191)

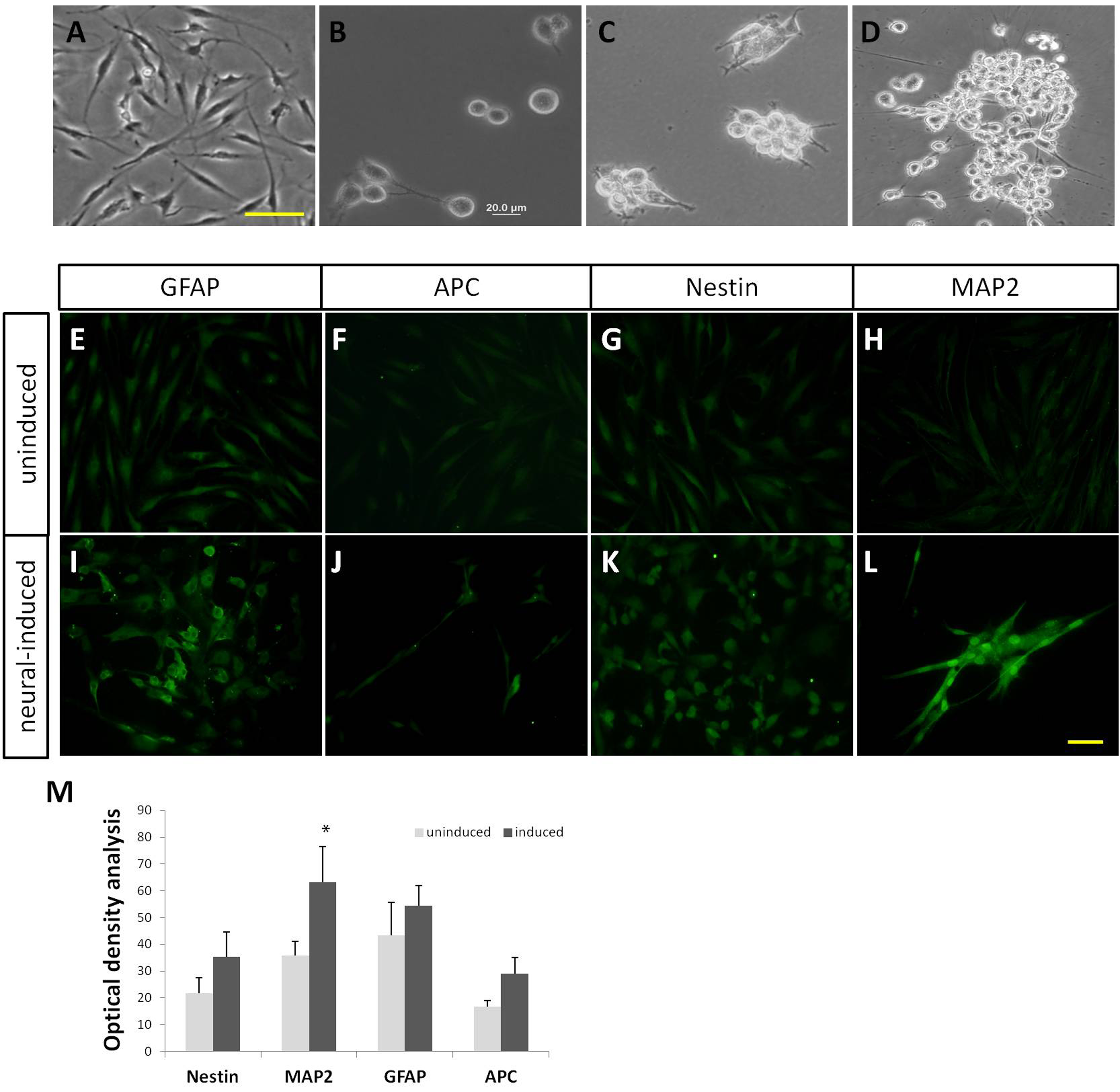

Supplement: Figure S1 — Neuroinduction of hEASCs (A): Morphology of uninduced hEASCs at p8 (B): Morphology of neural-induced hEASCs after 4 days at p8. (C, D): Morphology of hEASCs at p8 after neural-induced 10 days. The spherical floating aggregates were observed. (E–L) Immunocytochemistry of hEASCs before and afer neuroinduction at p8 (GFAP, APC, Nestin, MAP2). (M) Optical density analysis of immunocytochemistry before and after neuroinduction. Scale bar = 50 μm (A), 20 μm (B), 50 μm (E–L). Abbreviations: p, passage. [file jcmm0018-0326-sd1.tif]

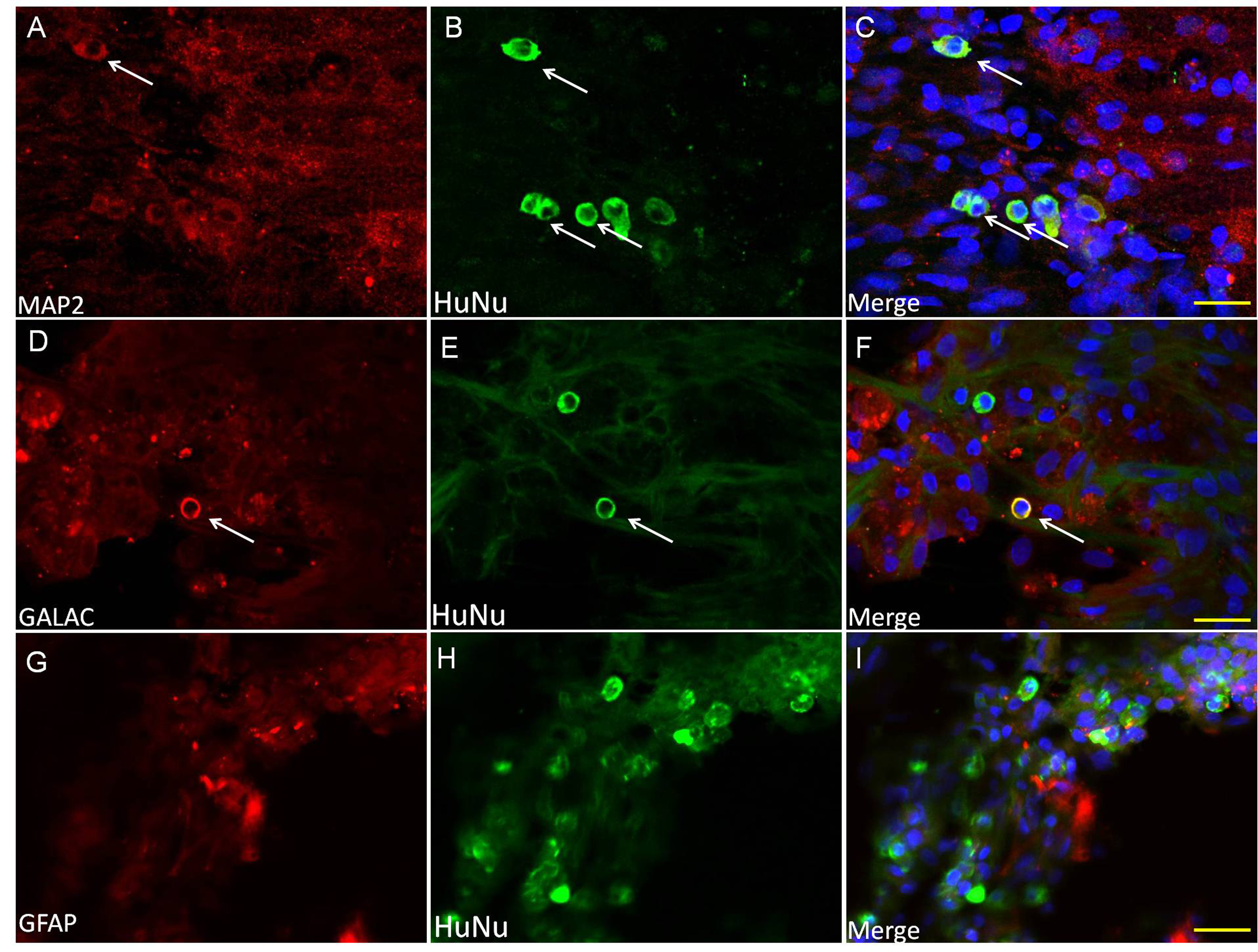

Supplement: Figure S2 — The fate of surviving hEASCs after 6 weeks in vivo. (A–F): Cells co-labelled with anti-HuNu (green) and mature neuronal marker MAP2 antibodies (red)/the mature oligodendrocyte marker GALAC (red) were detected within the white matter region. (G–I): No cells colabelled with anti-HuNu and GFAP antibodies were observed at 6 weeks after transplantation. These results showed that hEASCs have the potential to differentiate into neurons and oligodendrocytes, but not astrocytes. Nuclei are stained by 40,6-diamidino-2-phenylindole (DAPI). Scale bar = 20 μm (A–I) [file jcmm0018-0326-sd2.tif]

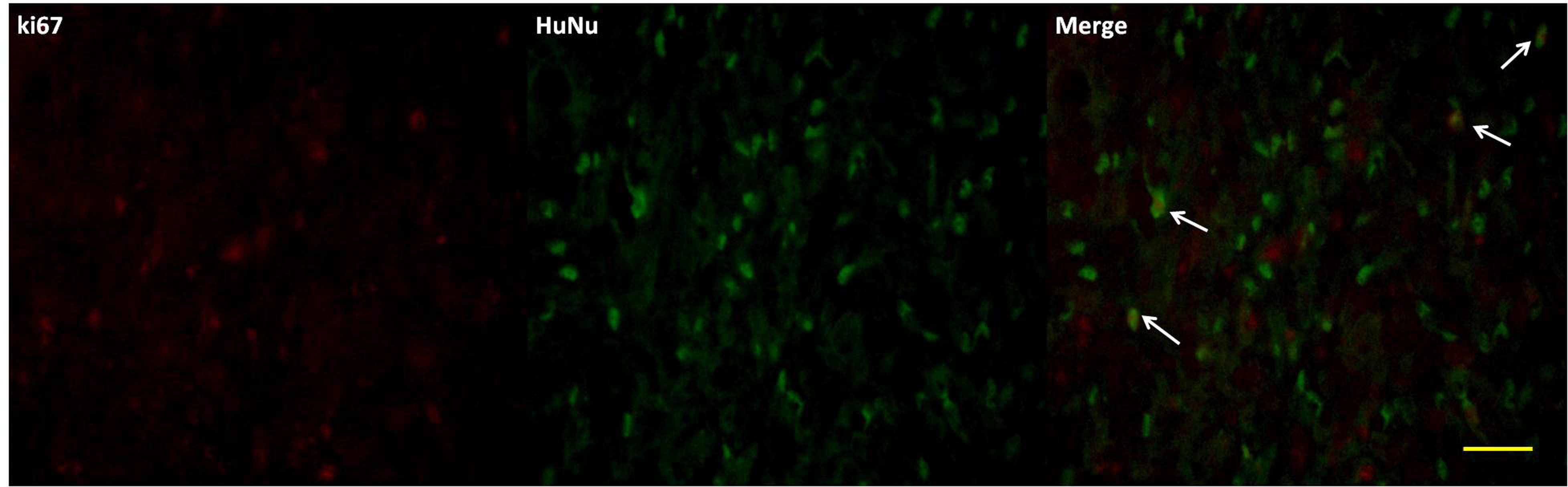

Supplement: Figure S3 — Anti-Ki67 and -HuNu Immunofluorescence of hEASCs after 6 weeks in vivo. [file jcmm0018-0326-sd3.tif]
